# Supplementary material for: Screening of Fungi for Potential Application of Self-Healing Concrete
Source: Sci Rep. 2019 Feb 14;9:2075. doi: 10.1038/s41598-019-39156-8 (PMC6375922; doi:10.1038/s41598-019-39156-8)
Supplement: Supplementary file 1 — Supplementary Information [file 41598_2019_39156_MOESM1_ESM.pdf]

Supplementary Information for

"Screening of Fungi for Potential Application of Self-Healing Concrete"

Rakenth R. Menon[1], Jing Luo[2], Xiaobo Chen[3], Hui Zhou[3], Zhiyong Liu[4], Guangwen Zhou[1,3], Ning Zhang[2,5*], Congrui Jin[1,3*]

1 Department of Mechanical Engineering, Binghamton University, NY 13902, USA

2 Department of Plant Biology, Rutgers University, New Brunswick, NJ 08901, USA

3 Materials Science and Engineering Program, Binghamton University, NY 13902, USA

4 Center for Neurodegeneration and Experimental Therapeutics, Department of Neurology, University of Alabama at Birmingham, Birmingham, AL 35294, USA

5 Department of Biochemistry and Microbiology, Rutgers University, New Brunswick, NJ 08901, USA

**Corresponding author:* ningz@rutgers.edu; cjin@binghamton.edu

Identification of the Yeast Strain

The yeast strain was obtained by culturing and purifying Active Dry Yeast (Hodgson Mill, Effingham, IL, USA) on PDA (Difco, BD Diagnostic Systems, Sparks, MD, USA). The purified fungal culture was grown on PDA for 7 days. Genomic DNA was extracted from the yeast cells with UltraClean Soil DNA Isolation Kit (MoBio Laboratories, Carlsbad, CA, USA). The nuclear ribosomal internal transcribed spacer (ITS) region, the universal fungal barcode marker, was amplified using the primers ITS1 and ITS4. PCR was performed with Taq 2X Master Mix (New England BioLabs, Maine, MA, USA). PCR cycling conditions for the ITS consisted of an initial denaturation step at 95 ^{o}C for 3 min, 35 cycles of 95 ^{o}C for 45 s, 52 ^{o}C for 45 s, 72 ^{o}C for 1 min and a final extension at 72 ^{o}C for 5 min. PCR products were purified with ExoSAP-IT (Affymetrix, Santa Clara, CA, USA) and sequenced with the PCR primers by Genscript, Piscataway, NJ, USA. The fungal strains were identified based on the search results of ITS sequences with BLASTn in GenBank as well as the morphological data.

Preparation of Mortar Specimens and Cement Paste Specimens

Series of mortar specimens were prepared for the survival test of the fungi in the environment of concrete by using Ordinary Portland Cement (CEM I 52.5N), standardized sand (DIN EN 196-1 Norm Sand) and tap water. The water-to-cement weight ratio was 0.5 and the sand-to-cement weight ratio was 3. The specimens were made according to the standard procedure NBN EN 196-1. They were then poured into 60 mm Petri dishes (9 ml per dish) and cured at 100% relative humidity and 22 ^{o}C for 28 days. Cement paste specimens were also prepared to investigate the pore size distribution of aging specimens. Ordinary Portland Cement (CEM I 52.5N) was mixed with tap water in a water-to-cement weight ratio of 0.5. Liquid paste was poured in molds with dimensions of 4 cm × 4 cm × 4 cm and cured at 100% relative humidity and 22 ^{o}C for 28 days. In addition, air-entrained cement paste specimens were also prepared to investigate the effect of air-entraining on the pore size distribution of the specimens. Eucon AEA-92 (Euclid Chemical, Cleveland, OH, USA) was dosed at a rate of 150mL per 100 kg of the total cementitious material.

Fungal Growth in the Harsh Environment of Concrete

The fungal growth in each type of plate has been shown in Fig. S1. Optical microscopic analysis of each case has been shown in Fig. S2. On the concrete plates, only one type of pH regulatory mutants of *Aspergillus nidulans*, i.e., MAD1445, has been found to be able to grow well. At 30 ºC, its growth rates reached 3.2 mm/day in the case of CMPDA30. Abundant conidia were observed from the plates with concrete and had similar morphology compared to those produced on the plates without concrete, as shown in Fig. S3.

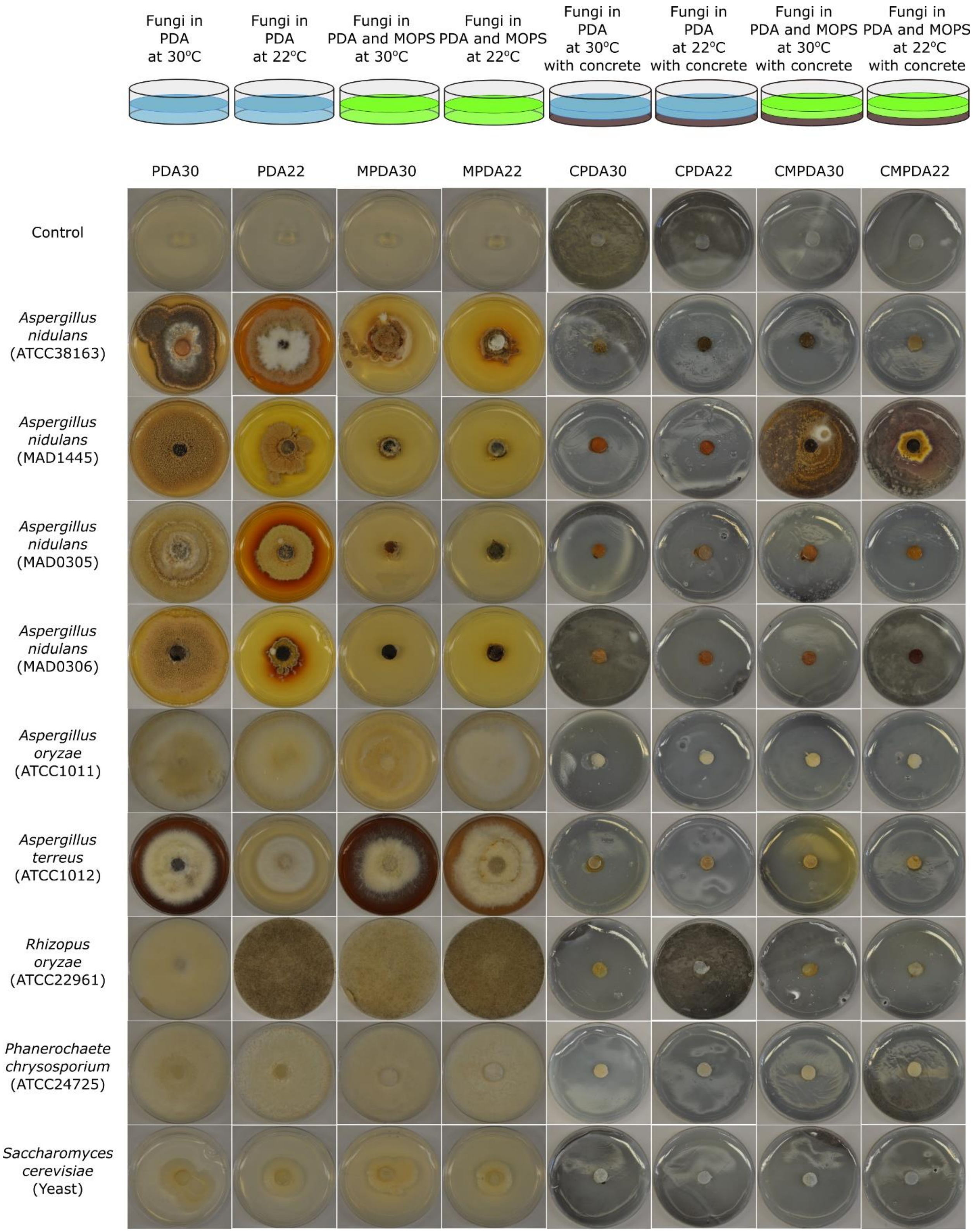

Figure S1. Only one type of pH regulatory mutants of A. nidulans, i.e., MAD1445, has been found to be able to grow well on the plates with concrete. In comparison, the other eight species did not grow on concrete.

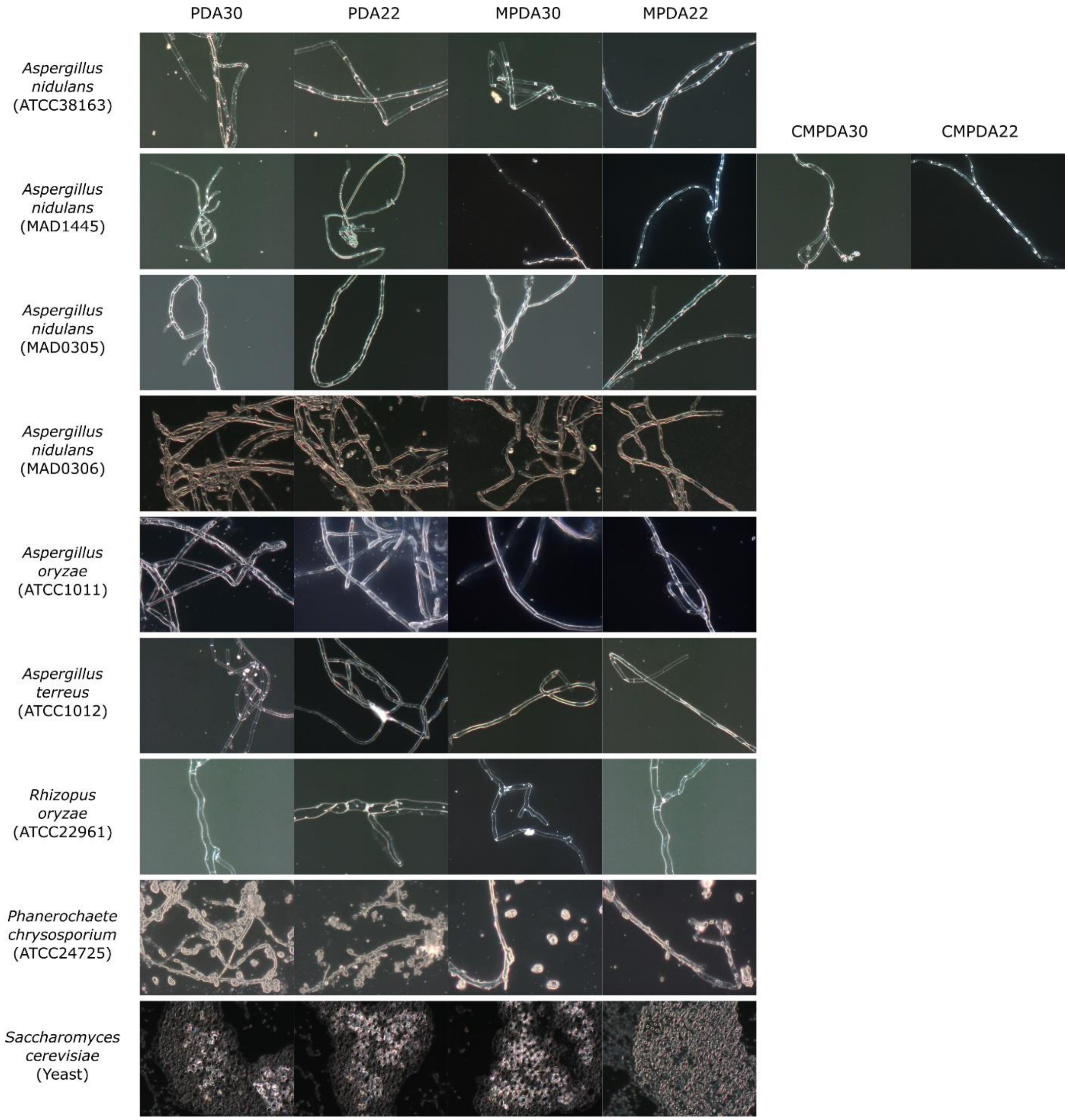

Figure S2. Microphotographs of optical microscopy (1000X, Carl Zeiss model III) confirmed that only one type of pH regulatory mutants of A. nidulans, i.e., MAD1445, was able to grow well on the plates with concrete.

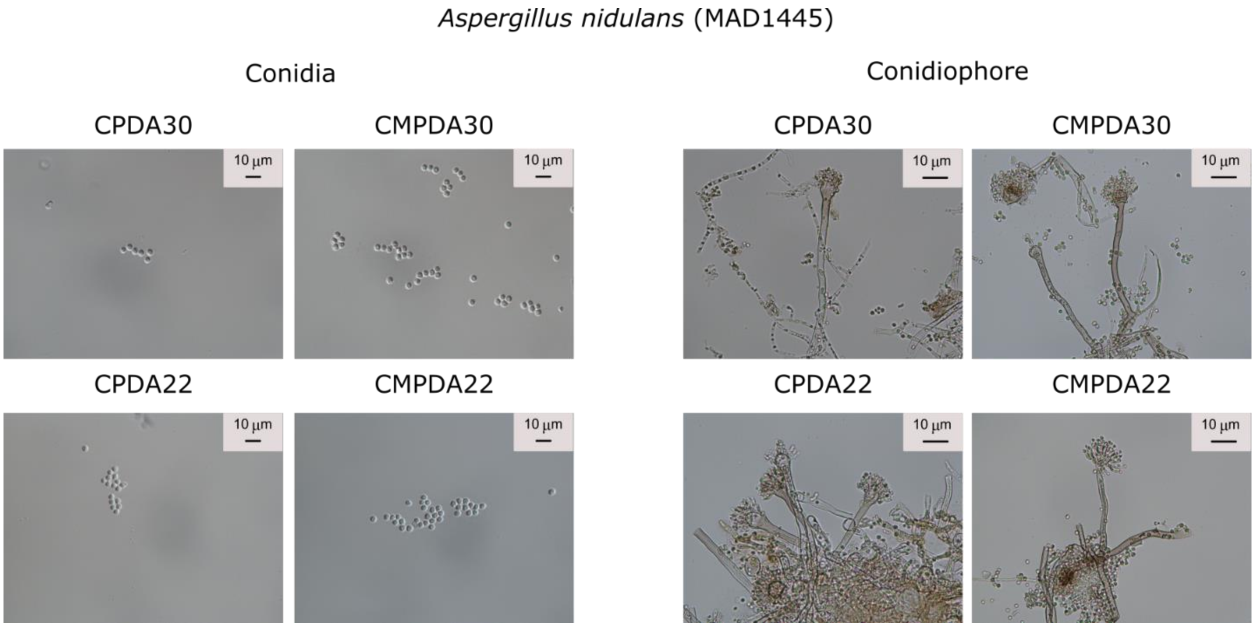

Figure S3. For the case of A. nidulans (MAD1445), abundant conidia were observed from the plates with concrete, which had similar morphology compared to those produced on the plates without concrete. The diameter of A. nidulans spores (round to oval in shape) appeared to be within the range of 3 μm to 4.5 μm.
